# Autochthonous and Probiotic Lactic Acid Bacteria Employed for Production of “Advanced Traditional Cheeses”

**DOI:** 10.3390/foods8090412

**Published:** 2019-09-13

**Authors:** Vincenzina Fusco, Grazia Marina Quero, Palmiro Poltronieri, Maria Morea, Federico Baruzzi

**Affiliations:** 1 Institute of Sciences of Food Production, National Research Council of Italy (CNR-ISPA), via G. Amendola 122/O, 70126 Bari, Italymaria.morea@ymail.com (M.M.); federico.baruzzi@ispa.cnr.it (F.B.); 2 Stazione Zoologica ‘Anton Dohrn’ of Naples, Integrative Marine Ecology Department, Villa comunale, 80121 Naples, Italy; 3 Institute of Sciences of Food Production, National Research Council of Italy (CNR-ISPA), via Monteroni, 73100 Lecce, Italy; palmiro.poltronieri@ispa.cnr.it

**Keywords:** *Streptococcus macedonicus*, *Lactobacillus rhamnosus*, *Lactococcus lactis*, autochthonous starter cultures, goat milk, cow milk, fresh cheese, ripened cheese, probiotic cheese, microbial food quality and safety

## Abstract

Microbial characterization of two Italian traditional cheeses, Giuncata and Caciotta Leccese, was carried out, with the aim to isolate autochthonous bacterial strains to be used as starters to improve and standardize the quality of these cheeses. More than 400 bacterial isolates were found, using PCR-based identification, to belong to 12 species of the *Streptococcus*, *Lactococcus*, *Lactobacillus*, and *Leuconostoc* genera. The dominant strains were screened for antagonistic activity against pathogenic and spoilage bacteria and exopolysaccharide production, acidification, and proteolytic activity. Since *Streptococcus macedonicus* was found to be the most prevalent lactic acid bacteria species present in milk and in both types of cheese, the best performing strain of this species was successfully used, alone or in combination with a selected autochthonous *Lactococcus lactis* strain, in pilot-scale productions of Giuncata and Caciotta Leccese cheeses, respectively. The combined inoculums of selected autochthonous strains positively influenced the sensory characteristics of both Giuncata and Caciotta cheeses. Finally, the selected autochthonous cultures were enriched with a potentially probiotic *Lactobacillus rhamnosus* strain and successfully used in pilot-scale productions of these traditional cheeses. To the best of our knowledge, this is the first study reporting the use of an autochthonous *S. macedonicus* strain as a starter for the production of cheeses with added probiotics. In addition, the identification of the probiotic strain in the feces of healthy volunteers fed with the advanced traditional cheese proved its effectiveness as a carrier for the delivery of probiotics to the human body.

## 1. Introduction

“Caciotta Leccese” is a semi-hard table cheese made with a blend of whole raw sheep, goat, and cow milk, falling in the large family of typical agrofood Italian caciotta cheeses such as “Caciotta della Lunigiana”, “Caciotta della Sabina”, “Caciotta di pecora Toscana” and the Protected Designed Origin (PDO) “Caciotta d’Urbino”. The milk blend is briefly fermented with thermophilic starters, renneted, and molded into 1.2–1.5 kg pieces. The cheese is then stewed at 37 °C for 2–4 h until it reaches pH 5.2, brined, and ripened approximately for two months. At the end of aging, Caciotta Leccese presents as a small-eyed, soft, yellowish paste with a mild to tangy flavor. As already reported [1], uncontrolled fermentation can blow, soften, or produce off flavors in this kind of cheese, so the use of adjuncts of selected mesophilic lactobacilli has been suggested in order to shorten the time of maturation and prevent eventual defects from occurring during ripening.

Giuncata Leccese is an un-ripened fresh cheese made from a blend of pasteurized sheep, cow, and goat milk. Giuncata is produced with milk heated to 72 °C, cooled to 32–38 °C, and coagulated in 25–30 min with the addition of calf or lamb liquid rennet without addition of a microbial starter. The curd is collected and drained into molds. Giuncata has no rind and the dough is white, with a soft and consistent texture, a mild acidic flavor, and a fine and intense odor of milk. As with other fresh cheeses, Giuncata is susceptible to the growth of spoilage microorganisms due to its oxygen content, high water activity, and neutral pH values [2]. Thus, in refrigerated storage, Giuncata has a shelf-life of only five days. However, inadequate hygienic conditions as well as occasional cold-chain interruptions may affect the final quality of this cheese, resulting in whey separation, increasing sourness and bitter taste. Thus, manufacturers need to standardize the production process of these traditional cheeses while still preserving their taste and sensory quality. To achieve these goals, the selection and use of autochthonous microbial strain cultures seems to be the best solution, as has already been reported [3,4,5]. Moreover, given the increasing demand for foods providing consumer health benefits, probiotic cultures [6] are being extensively exploited to add functional properties to traditional cheeses. In addition, these studies have also aimed to increase the market competitiveness of these cheeses, which has traditionally been based only on their sensory characteristics. However, as discussed by Di Lena et al. [7], being strain-specific and affected by the intrinsic characteristics of each probiotic-delivering matrix, the survivability and functionality of a given probiotic strain should be assessed not only during the production and storage of each specific food, but also during digestion of the resulting probiotic food.

Herein, we have characterized the microbial populations of Giuncata and Caciotta Leccese cheeses. Since *S. macedonicus* was found to be the most prevalent non-starter lactic acid bacteria (LAB) species present in the milk and in both types of cheese, the best performing strain of this species was used, alone or in combination with a *Lactococcus lactis* strain, as (multiple) autochthonous strain culture (ASC) to standardize cheese processing. Once this goal was achieved we coupled both ASC-based cheese productions with the addition of a probiotic *Lactobacillus rhamnosus* strain and investigated its behavior during production, ripening, and shelf-life of both cheeses, as well as its survival in the feces of healthy volunteers.

## 2. Materials and Methods

In order to characterize the microbiota of Caciotta and Giuncata Leccese cheeses, several samples were taken from different dairies in the same region. The isolation and characterization of the dominant strains allowed selection of autochthonous (multiple) starter cultures (ASCs). Several lab- and pilot-scale production runs of both cheeses were carried out to define the best ASC for each type of cheese. Once ASCs were defined, they were supplemented with a commercial probiotic strain and used for the production of “advanced” traditional Caciotta and Giuncata Leccese cheeses.

### 2.1. Cheese-Making and Sampling

Investigations were carried out on eight Giuncata and eight Caciotta Leccese cheeses manufactured in six farmhouses located in the Salento (Apulia region, Italy), following the traditional protocols described below.

Three batches of each type of cheese were also manufactured (using the selected autochthonous starter cultures) at the dairy farm “L’Aia Vecchia S.r.l.”, located in the same geographic area (Pisignano, Vernole, Lecce, Italy) and well known for its traditional production technology and premium quality production.

Raw milk used for manufacturing both cheeses was a blend of sheep, goat, and cow milk; it was filtered with a stainless steel filter (1 mm cut-off), and promptly heated to 72 °C for 15 s; thereafter, for manufacturing Giuncata, it was transferred into the coagulation tank and cooled to 34 °C, salt (1.2% *w/w*) and approximately 20 g/q of liquid calf rennet (Caglificio Clerici, Como, Italy) were added, and the cheese was kept at this temperature to allow curd sedimentation over approximately 20 min. Curd was then broken into "nut-sized pieces", collected, and drained into molds (reed containers called “giunco”, hence the name “Giuncata”) at 0–2 °C. For Caciotta Leccese, the pasteurized milk blend was cooled to 37–38 °C, and after addition of either commercial (Sacco, Cadorago, Como, Italy) starters of *Streptococcus* (*S.*) *thermophilus* and *Lactobacillus delbrueckii* subsp. *lactis* (initial cell density for each species was approximately 9.0 ± 0.2 log CFU mL^−1^; control cheese, CC) or the selected autochthonous starters (*S. macedonicus* 62GT0 and *Lactococcus* (*L.*) *lactis* LC51 at final concentrations of approximately 10^5^ and 10^6^ CFU/mL, respectively) and liquid calf rennet (Caglificio Clerici), was kept at the same temperature for approximately 20 min to allow curd sedimentation. The curd was then broken into grains, transferred into riddled molds, pressed to drain the whey, transferred to an artisanal warm room at 23–26 °C (for the so-called *stufatura* (stewing) process) to reach a pH value of approximately 5.15, and then salted in brine (18 °Bé) for 10 h. Thereafter, Caciotta cheeses were ripened at about 10 °C and 85% relative humidity for 60 days.

The milk samples were collected directly from the vat prior to rennet addition, and the curd samples were collected after the molding. Caciotta and Giuncata cheeses were collected as well. All samples were collected using the standard method [8]. Samples were kept on ice in isothermal bags during their transfer to CNR-ISPA (National Research Council of Italy, Institute of Sciences of Food Production) laboratories, where they were immediately analyzed.

### 2.2. Enumeration and Isolation of Microorganisms and Preliminary Characterization of Isolates

Twenty-five mL or g of each sample were dispersed in 225 mL of Quarter Strength Ringer’s Solution (Oxoid S.p.A., Garbagnate, Milan, Italy), homogenized, and serially diluted in sterile 0.1% buffered peptone water [9]. The appropriate dilutions were plated in triplicate on: (i) plate count agar (PCA, Oxoid S.p.A.) for counting of total aerobic bacteria, (ii) M17 (Oxoid) with 0.5% lactose (LM17) for Gram-positive cocci; (iii) Rogosa (Oxoid) or de Man, Rogosa and Sharpe (MRS) (Oxoid) for mesophilic Gram-positive bacilli; (iv) violet red bile agar (VRBA, Difco Laboratories, Detroit, MI) for coliforms; (v) potato dextrose agar (PDA, Difco) supplemented with chloramphenicol (0.1 g/L) for yeasts and molds and Baird Parker (BP, Oxoid) or mannitol salt agar (MSA, Oxoid) for staphylococci. PCA plates were incubated at 30 °C for 24 h, and LM17 and MRS dishes at 42 and 30 °C, respectively, for 48 h under anaerobic conditions (AnaeroGene, Oxoid S.p.A); VRBA and BP or MSA were incubated at 37 °C for 48 h and PDA plates at 25 °C for 7 days. Microbial analyses were performed in triplicate. Isolated colonies were randomly picked up from the highest plate dilutions of MRS, Rogosa, and LM17 agar and purified by repeated streaking on the relevant media. All isolates were characterized by Gram staining, catalase activity, spore formation, and gas production from glucose by the hot loop test [10]. All isolates were stored at −80 °C in MRS broth added with 20% sterile glycerol.

### 2.3. Genomic DNA Extraction and Molecular Analysis of Isolates

DNA isolation and purification was carried out as previously reported [11], assessing its purity and quantity as reported by Fusco et al. [12].

Cocci-shaped lactic acid bacteria (LAB) were identified by amplifying the intergenic spacer region between 16S and 23S rRNA genes, as described by Jensen, Webster, and Straus [13], and as reported by Quero et al. [5]. In order to achieve their speciation, isolates providing a unique class of 360 bp spacer were further subjected to species-specific polymerase chain reaction (PCR) for *Streptococcus* (*S.*) *thermophilus* [14], and *S. macedonicus* [15], using the protocol described by Coppola et al. [16]. Rod-shaped LAB were referred to the genus *Lactobacillus* by means of 16S–23S rDNA spacer analysis [13], and were identified at a species level using the protocol described by Aponte et al. [17] using the PCR-RFLP approach described by Blaiotta et al. [18]. RAPD-PCR of isolates was performed as described by Baruzzi et al. [19].

### 2.4. Technological Characterization of Bacterial Strains

All strains belonging to the dominant species were screened for their main pro-technological activities such as exopolysaccharide production, citrate metabolism, acidification, proteolytic and lipolytic activities, and antagonistic activity against pathogenic and spoilage bacteria. In particular, the capability to produce exopolysaccharides (EPS) was assayed according the method of Dabour and LaPointe [20] with slight modifications. Selected LAB strains were streaked with a sterile toothpick on MRS or M17 agar plates, containing 0.08% ruthenium red and in which glucose was replaced with 5% of one of the following sugars: fructose, galactose, glucose, lactose, or saccharose (Sigma Aldrich, Milan, Italy). Plates were incubated at 30 °C for 72 h under anaerobic conditions (Anaerogen kit, Oxoid). To distinguish EPS-producing colonies, capsular polysaccharide (CPS) production by mucoid colonies was further assayed by Indian ink staining method [21].

Citrate metabolism was screened as previously reported by Kempler and McKay [22].

The acidification activity in reconstituted skim milk of the selected LAB strain cultures was evaluated by pH measurement using a pH meter (Oakton Bench top pH 510 Meters, Cole-Parmer, Vernon Hills, IL, USA). Briefly, 1% (approximately 6 log CFU/mL) of each strain culture was inoculated into 9% skim milk pasteurized at 90 °C for 20 min. The analysis was carried out in triplicate using non-inoculated skim milk as a negative control. The pH values were registered at time 0 and after 3 and 24 h of incubation at 37 °C. Multiple strain cultures (in skim milk), composed of combinations of three strains (1% i.e., approximately 6 log CFU/mL of each strain), were also evaluated by pH measurement, as reported above.

Proteolytic activity of the autochthonous isolates was analyzed by applying the protocol of Church et al. [23], as described for lactic acid bacteria growing in milk [24], and their lipolytic activity was tested following the protocol of Lamy and Galaup [25].

The antagonistic activity of the indigenous strains against pathogenic and spoilage microorganisms was evaluated by the deferred method [26]. *Staphylococcus* (*Staph*.) *aureus* ATCC14453, *Listeria monocytogenes* DSM20600, *E. coli* K12, *Staph*. *epidermidis* DSM20044, and *Streptococcus* (*S.*) *parauberis* 6GT5 (isolated from Giuncata stored at 4 °C for 5 days) were used as indicator strains. Briefly, overnight broth cultures in MRS broth of each LAB strain were centrifuged at 7000 rpm for 7 min; pellets were then washed and resuspended in sterile 0.5% NaCl solution. The resulting cell suspensions were spotted on MRS agar plates and incubated for 24 h at 37 °C to allow colonies to develop. The overlay of tryptic soy agar (TSA; Oxoid S.p.A.) soft agar was inoculated with exponential cells of each indicator microorganism and poured onto the plates. Colonies of the producer strains were checked for clear zones of inhibition around them after incubation at 37 °C for 24 h.

In order to avoid the spreading of antibiotic resistance through the use of an authocthonous starter culture (ASC), the best performing strains isolated from samples of Giuncata and Caciotta throughout their production and ripening were analyzed for the presence of resistance factors to some antibiotics, applying the disk diffusion method developed by the British Society for Antimicrobial Chemotherapy [27] using, when necessary, cut-offs of the values proposed by Tosi et al. [28]. The assays, performed for 24 h, involved testing for antibiotic resistance to ampicillin, chloramphenicol, tetracycline, gentamicin, clindamycin, kanamycin, streptomycin, and erythromycin.

### 2.5. Pilot-Scale Cheese-Making of Giuncata and Caciotta Leccese with Autochthonous and Probiotic Strains

Both Caciotta and Giuncata Leccese cheeses were produced at L’Aia Vecchia S.r.l. dairy plant (Vernole, Lecce, Italy), using the selected autochthonous strain cultures (ASC), in triplicate, following the traditional protocol described in Section 2.1. In particular, the ASC2 composed of the *S. macedonicus* 62GT0 strain (final density of approximately 10^5^ CFU/mL in 300 L of milk for each of the three batches) was used to produce Giuncata Leccese cheese, while the ASC4 composed of *S. macedonicus* 62GT0 and *L. lactis* LC51 (final density of approximately 10^5^ and 10^6^ CFU/mL, respectively, in 100 L of milk for each of the three batches) was used to produce Caciotta Leccese cheese.

“Advanced” Giuncata and Caciotta Leccese cheeses were made from pasteurized milk to which, besides the technological starter, the biomass of a probiotic commercial strain was added. This latter strain, isolated from the human gastrointestinal tract, deposited in the Leibniz Institute DSMZ-German Collection of Microorganisms and Cell Cultures GmbH as *Lb. rhamnosus* DSM 16605 and sold as an ingredient for food products and/or supplements, is already on the market as a freeze-dried product named Serilac–LR04.

In particular, for both cheeses, the technological starter was added at the final density of approximately 10^5^ CFU/mL in 100 L of milk for each of the three batches, and the probiotic strain at the final density of 10^6^ CFU/mL in 100 L of milk for each of the three batches.

Samples of Giuncata were taken after 0, 1, and 7 days of storage at 4 °C, whereas for each type of Caciotta Leccese produced, milk (inoculated and uninoculated), curd, and Caciotta samples after 0 and 60 days of ripening were collected, transferred on ice to CNR-ISPA laboratories, and immediately analyzed. Giuncata and Caciotta cheeses without the adjunct strains were also used and sampled as controls. Aliquots of 10-fold dilutions in Quarter Strength Ringer’s Solution (Oxoid) (ISO, 2001) of each sample were plated on maltose-MRS-vancomycin (MMV), selective for the strains of the *Lb. casei* group [7], on LM17 for *L. lactis*, and on the substrate SM, selective for *S. macedonicus* [29]. Plates were incubated at 30, 10, and 42 °C, respectively, to promote the growth and detection of the strains used as starters. In order to monitor the pro-technological ASC2 and ASC4 cultures from cheese samples, colonies isolated from the plates containing the highest dilutions were purified and subjected to DNA extraction and quantification [11,12], 16S–23S rDNA spacer analysis (to detect *L. lactis*), *S. macedonicus*-specific PCR [15], *Lb. rhamnosus* specific PCR [30], and RAPD-PCR and REP-PCR as described by Di Lena et al. [7].

### 2.6. Sensory Analysis

A panel of six panelists trained in the sensory analysis of cheeses produced in Apulia region (Southern Italy), evaluated samples of Giuncata refrigerated for 48 h and Caciotta ripened for 2 months, produced using the autochthonous strain cultures (ASC), and the same cheeses also amended with the probiotic *Lb. rhamnosus* strain. The control cheeses, manufactured without the adjunct strains, were also evaluated. Panelists were asked to assess the following specific sensory descriptors for taste and texture: flat (samples tasteless or without distinct or dominant sensory notes), sweet (samples producing stimuli on the tip of the tongue as sugar solutions), acidic (samples having sharp, tart, or sour taste, as yoghurt or vinegar) and bitter (samples producing stimuli in the inner part of the tongue as produced by caffeine in water), creamy (samples with a smooth texture, as of cream), soft (samples simple to chew that do not produce any pointed particles), gummy (samples that need to be chewed at least five times before being swallowed), hard (samples that need a strong bite to be chewed, as raw carrots or nuts). Sensory analysis was performed following the methodology reported by Franciosi et al. [31]. This method was applied only to verify the occurrence of appreciable differences in sensory descriptors between different types of the same cheese. Thus, the result of this paired qualitative comparison test gave preliminary information about the overall acceptability of the new cheeses that could not be subjected to statistical analysis.

### 2.7. Advanced Cheese Feeding Procedure

Since the viability of probiotic bacteria may be affected during cheese manufacturing and storage, as well as during digestion of the probiotic cheese by a human, a preliminary *in vivo* assay was carried out to ascertain the survival of the probiotic *Lb. rhamnosus* LR04 strain in the human gastrointestinal tract after cheese ingestion. Three volunteers, two males and one female (A, F, L), aged between 30 and 40 years, who had not received antibiotic therapy in at least the last two months and who had apparent good health, consumed advanced Caciotta Leccese cheese, ripened for 30 days, as follows:

Step 1: Volunteers avoided eating fermented foods containing lactic acid bacteria (yogurt, cheese, salami, etc.) for at least six days before the start of the trial (Day 0);

Step 2: Volunteers consumed approximately 100 g of the advanced cheese daily, containing approximately 10^9^ CFU/g of *Lb. rhamnosus* in a single dose, for 6 days (Day 6);

Step 3: Volunteers finished consumption of the advanced cheese, and avoided the consumption of other fermented foods for 3 days following Step 2 (Day 9).

The study was conducted in accordance with the Declaration of Helsinki. All subjects gave their informed consent for inclusion before they participated in the study.

Fresh fecal samples were processed as previously reported [32]. Briefly, fecal samples were suspended in anaerobic phosphate buffer (pH 7.0) at 10% (wet weight/volume); fecal slurry was then decimally diluted in the same buffer. Aliquots of each dilution were plated on MMV agar plates (Di Lena et al., 2015) and incubated at 37 °C in anaerobiosis. More than 70 colonies for each sample were isolated from the plates with the highest dilutions. DNA from colonies was isolated, purified, and quantified as described in Section 2.3.

Molecular monitoring of the probiotic strain was carried out by specific PCR [30], RAPD-PCR, and REP-PCR, following the protocols described by Di Lena et al. [7].

## 3. Results and Discussion

### 3.1. Isolation and Molecular Characterization of Microorganisms from Cheeses

The study of the ecosystem characterizing the transformation of milk in Giuncata and Caciotta Leccese cheeses made it feasible to identify the most representative microbial species occurring in these traditional productions.

The starting raw milk used to manufacture both cheeses presented a numerous and heterogeneous microflora, with a prevalence of lactic acid bacteria (LAB) accounting for more than 10^6^ log CFU/mL. Pasteurization of the milk caused a reduction of the microbial populations by approximately two decades. The prevalence of LAB, which are essential in cheese production, and the absence or low presence of spoilage and potentially pathogenic microorganisms such as enterobacteria, staphylococci, yeasts, and molds (responsible for spoilage) indicated good suitability of this milk for cheese-making.

In fresh Giuncata, lactobacilli and lactococci loads were retrieved at approximately 10^3^–10^4^ CFU/g, reflecting the composition of the milk in the vat. This was expected, since coagulation, shaping, and packaging, lasting approximately 30–45 min, if carried out under hygienic conditions, does not promote the sprouting and growth of microorganisms. Giuncata Leccese is normally withdrawn from the market one week from the date of processing and packaging, with an expiry date of 10 days. *Streptococcus parauberis* was the dominant species found in Giuncata after 10 days of storage at 10 °C. It is a common agent of bovine mastitis that is frequently found in cheeses as part of the undesired background microflora [33,34].

Based on its potential to spoil Giuncata cheese, a *S. parauberis* strain isolated from Giuncata was used as an indicator to test the antimicrobial activity of the selected strains.

**Figure 1:**
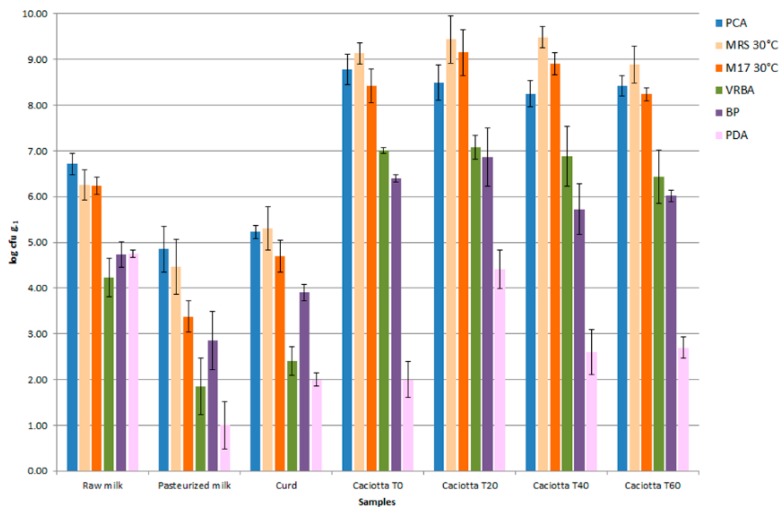
Distribution of microbial populations at different steps of traditional production of Caciotta Leccese.

Processes that characterize the later stages of coagulation, stewing, and ripening of Caciotta Leccese promoted the prevalence of a valid lactic microflora, and within this, the advantage of cocci on rods over anti-dairy microorganisms. The distribution of microbial populations at the different stages of the traditional production of Caciotta Leccese is shown in Figure 1.

The taxonomic identification of 300 isolates from samples collected during the different steps of the production process of both Giuncata and Caciotta was carried out, applying the molecular techniques reported in Section 2.3.

**Figure 2:**
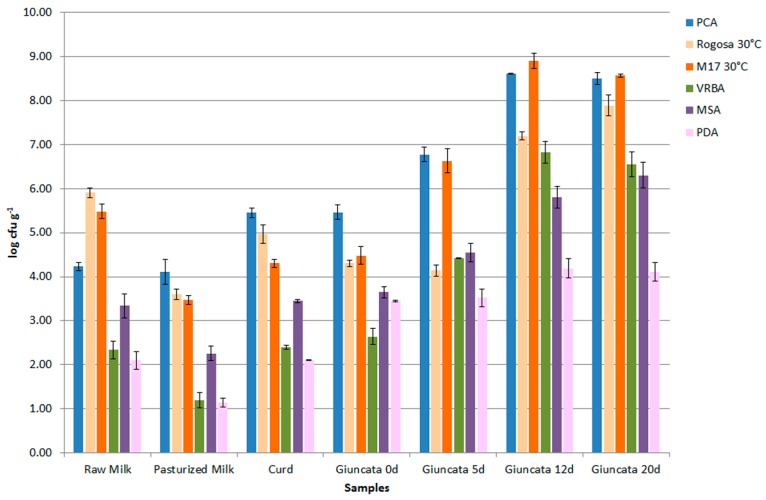
Distribution of microbial populations at different steps of traditional production of Giuncata Leccese, and during its storage at 10 °C for 20 days.

The main microbial populations of Giuncata Leccese (Figure 2) included the following species (in decreasing numerical order): *S. thermophilus*, *S. macedonicus*, *Lc. lactis* subsp. *lactis*, *Leuconostoc* (*Leuc*.) *mesenteroides*, *Lactococcus* spp., *Lb. casei*, *Lb. delbruekii* subsp. *lactis*, *Lb*. *plantarum*, *Macrococcus* (*Mc*.) *caseolyticus*, *S. parauberis*, and *Staphylococcus* spp., as detailed in Table 1.

**Figure 3:**
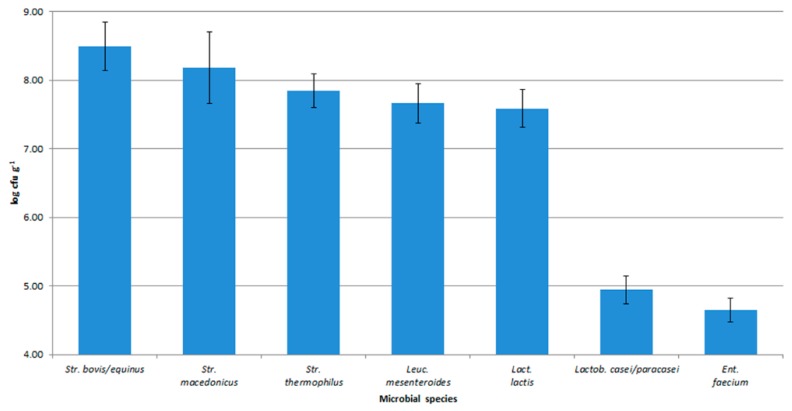
Distribution of endogenous species in Caciotta Leccese after 60 days of ripening.

In Figure 3, the distribution of species in Caciotta Leccese at 60 days of ripening is shown.

The taxonomic identification of the lactic acid bacteria collected during the production process of traditional Caciotta Leccese ascertained that *S. macedonicus* and *S. thermophilus* were the dominant species in this cheese. *S. macedonicus*, isolated at low loads from the starting raw milk, survived the pasteurization step, increased in the curd, and was dominant in the final product. Thus, this species could be considered the most representative of the technological ecosystems herein analyzed, and very probably responsible for conferring the typicality to the final product.

As concerns the distribution of species in the cold-stored final products, *Lc. lactis*, *S. parauberis*, *Lactococcus* spp., and *Leuc. mesenteroides* were the species most frequently found among cocci strains.

### 3.2. Technological Characterization of Bacterial Strains

Indigenous lactic acid bacteria, endowed with phenotypic traits particularly promising for their technological profile and appropriately selected might, when added to pasteurized milk in appropriate quali-quantitative ratios, favor associations and synergies that ensure the achievement of constant quality standards and requirements of hygiene, as well as ensuring the normal course of the production process of Giuncata and Caciotta Leccese.

For this purpose, autochthonous lactic acid bacteria strains found to be dominant in the various steps of Giuncata and Caciotta Leccese production were analyzed for some pro-technological traits (expoliysaccharide production, citrate metabolism, acidification, lipolytic and proteolytic activities, and inhibition of pathogens).

Among the hetero-fermenting lactic acid bacteria isolated from Giuncata, one *Leuc. mesenteroides* strain resulted ropy mucoid on MRS supplemented with 5% sucrose. Interestingly, among the homo-fermentative lactic acid bacteria isolated, eight *S. thermophilus* strains showed a ropy phenotype on a substrate supplemented with lactose. No *S. thermophilus* strains showed a mucoid appearance.

Eight autochthonous strains, four *Lc. lactis* and four *Leuc. mesenteroides,* resulted positive for citrate metabolism.

Antagonistic activity against pathogenic and spoilage microorganisms was evaluated by the deferred method [26] as described in Section 2.4. *Staph. aureus* ATCC14453, *Listeria monocytogenes* DSM20600, *E. coli* K12, *Staph. epidermidis* DSM20044, and *S. parauberis* 6GT5 (isolated from Giuncata stored at 4 °C for 5 days) were used as indicators. A total of 126 strains (4 *Leuc. mesenteroides*, 39 *S. thermophilus*, 25 *S. macedonicus*, 46 *Lc. lactis*, and 12 *Lactococcus* spp.) from samples collected during the different steps of cheese production, from the final products, and during their storage at 4 °C, were tested for antagonistic activity against the above mentioned indicator strains that could occur in milk and/or Giuncata and Caciotta Leccese cheeses. Four *Leuc. mesenteroides* and four *S. macedonicus* showed inhibition toward all the indicator strains, while the remaining ones showed antagonist activity against at least two of the indicator strains.

Lipolysis is very important for the flavor and aroma of cheeses. Normally, lactic acid bacteria are not lipolytic, even though an esterase activity can be developed by specific starter cultures. All the tested strains, analyzed as reported in Section 2.4, did not show any appreciable lipolytic activity.

Based on RAPD fingerprints and 16S rDNA sequence-based taxonomic identification, and taking into account their technological features, the strains listed in Table 2 were further characterized.

Based on the results obtained by the molecular and technological strain typing, the LAB listed in Table 2 were considered promising candidates to be used as starters, and were analyzed for the presence of resistance factors to some antibiotics, as reported in Section 2.4. On the basis of analysis of the diameters of the inhibition halos, all strains were devoid of antibiotic resistances or showed values not different from those of the commercial starter stored in the international microbial collections.

**Figure 4:**
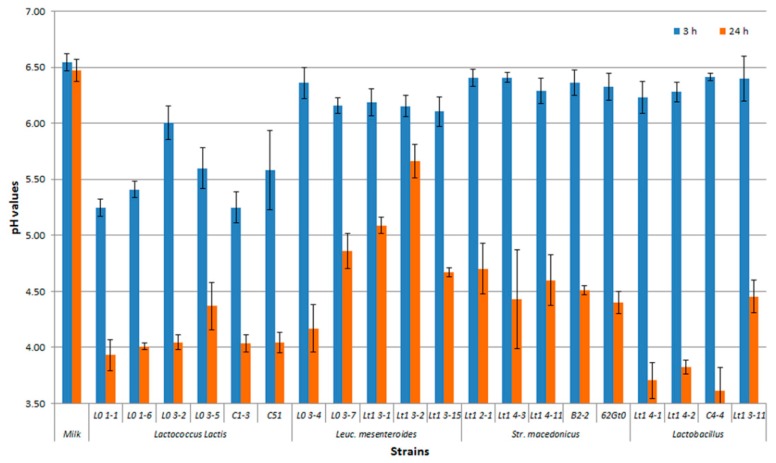
Values of pasteurized milk acidification of selected autochthonous lactic acid bacteria tested.

In Figure 4, the results of the acidification test on the selected autochthonous strains are shown. *Lb. delbrueckii* subsp. *bulgaricus* and most *Lc*. *lactis* strains were endowed with the best acidification activities. The fastest strains, able to reduce the pH values within three hours after the inoculum, were lactococci, although lactobacilli were the most acidifying after 24 h (Figure 4). A low acidifying activity was found in the *Leuconostoc* and *Streptococcus* strains tested.

**Figure 5:**
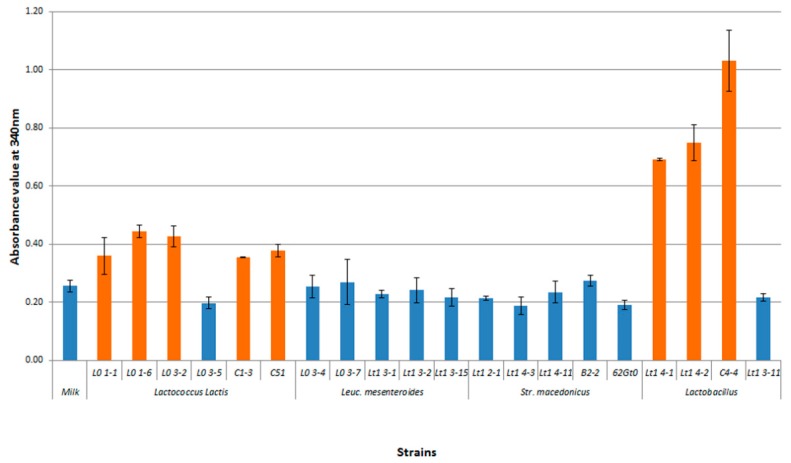
Proteolytic activity of some of the autochthonous lactic acid bacteria tested. Orange bars represent significantly higher proteolytic activity values.

The most representative results of the analysis of the proteolytic activity of selected lactic acid bacteria are reported in Figure 5. Most strains did not develop significant proteolytic activities. In particular, no *Leuc. mesenteroides* and *S. macedonicus* strains produced absorbance values different from those of the control. *Lb. delbrueckii* subsp. *bulgaricus* showed the most proteolytic activity, while all the strains of *Lc. lactis*, with the exclusion of L03-5, showed significant proteolytic activity (Figure 5).

The autochthonous strains endowed with phenotypic traits potentially suitable for the production of the cheeses under study, were used for the formulation of cultures. Mini-productions of cheese were carried out at a laboratory scale using different combinations of strains, as reported in Section 2.5.

*Lc. lactis* LC51, with good acidifying and proteolytic activities, and *S. macedonicus* 62GT0, which showed an antagonistic activity in *in vitro* inhibition assays against all tested indicator strains, were identified as valid and therefore useful for the realization of an autochthonous technological starter.

Samples of fresh Giuncata and cheese after 1 and 4 days of cold storage were analyzed as reported in Section 2.3. Table 2 shows the counts of the strains for the different samples analyzed.

The application of molecular techniques [13,15] made it feasible to confirm that all colonies isolated from the plates belonged to the *Lc. lactis* and *S. macedonicus* species used as starters.

In the case of the trial ASC3, coagulation was faster and the clot was more consistent, probably due to a synergistic action of the two strains. As demonstrated by viable cell counts (Table 3), the strains used as starters survived well either alone or together under the processing conditions applied.

The presence of high counts of coccus-shaped lactic acid bacteria, while being a preventive factor against the development of other spoilage microorganisms or eventually pathogens, led Giuncata to take on a sour taste with hints of maturity on the sixth day of cold storage. The organoleptic characteristics found were not unpleasant (due to high acidity, intense aroma, and “mature” taste of the cheese), but were simply not typical for this product. By contrast, this characteristic was absent when Giuncata was inoculated only with *S. macedonicus.* For this reason, the selected *Lc. lactis* strain was excluded as a starter for the production of Giuncata.

*S. macedonicus* 62GT0 was identified as valid and therefore useful as a technological starter for this fresh traditional cheese. This strain not only showed antagonistic activity against all tested spoilage and pathogenic bacteria in the in vitro inhibition assays, but also, when this strain was applied for manufacturing Giuncata, a low post-acidification was found in the cheese, with consequent extension of its shelf-life.

### 3.3. Experimental Pilot-Scale Making of Giuncata and Caciotta Amended with Pro-Technological Strains and with the Probiotic Strain

Six batches (three of Giuncata and three of Caciotta Leccese) were manufactured at L’Aia Vecchia S,r,l., as reported in Section 2.6 to standardize their production while maintaining the typicality of the cheeses. In particular, the starter for Giuncata was composed only of *S. macedonicus* 62GT0 (ASC2; final density of approximately 10^5^ CFU/mL in 300 L of milk for each of the three batches), and that for Caciotta was composed of the selected autochthonous strains of *S. macedonicus* 62GT0 and *Lc. lactis* LC51 (ASC4; final density of approximately 10^5^ and 10^6^ CFU/mL, respectively, in 100 L of milk for each of the three batches). Additionally, advanced traditional Giuncata and Caciotta Leccese cheeses were produced in triplicate, with addition to the autochthonous starters of the commercial probiotic *Lb. rhamnosus* LR04 (final density of 10^6^ CFU/mL in 100 L of milk for each of the three batches).

### 3.4. Sensory Analyses

The combined inoculums of the selected autochthonous strains were judged to have positively influenced the sensory characteristics of both Giuncata and Caciotta cheeses, since they were well appreciated by expert tasters. Results of the paired comparison test, based on a qualitative assessment of sensory descriptors, are shown in Table 4 and Table 5.

Giuncata leccese is an unfermented fresh cheese refrigerated immediately after curdling. For this reason, its “flat” taste is the same as the milk. The ASC samples still presented a milky taste, although some additional flavor notes were perceived by assessors. These differences could be due to the slow acidification produced by the ASC culture during its 48 h of refrigeration, resulting in a weak sour flavor. It is quite interesting that no bitterness was reported. The inoculation of ASC did not change any texture descriptor considered here.

Caciotta leccese is a full-fat, soft, medium-ripened cheese. In this work, control cheese was produced using commercial starter mainly to acidify curd during the stewing step. By contrast, the addition of the ASC had the intended purpose of improving ripening and conferring distinctive organoleptic characteristics to this cheese. In contrast to the Giuncata leccese, in this case the “flat” taste was considered usual for the soft ripened cheese. The ASC samples produced some flavor notes different from the CC samples. Based on taste descriptors, differences in flavor notes could have been due to the reduction of acid and bitter sensory notes. However, ASC samples were differentiated by CC samples more in the texture descriptors: in fact, ASC samples were found to be generally more creamy and soft.

In order to produce an advanced traditional cheese, ASC was supplemented with the commercial probiotic *Lb. rhamnosus* LR04 strain. It is worthy of note that no differences were found in sensory analysis of Giuncata cheese with ASC, whereas advanced Caciotta leccese cheese samples containing both ASC and *Lb. rhamnosus* LR04 were found to be more soft and “cheesy” (taste and smell of ripened cheese) than their respective ASC samples. No other differences were perceived.

It is worth mentioning that the sensory assessment was carried out only for preliminary information about possible changes in the main sensory descriptors of traditional cheeses due to changes in the starter formulation. The absence of defects, as well as the small improvement in some sensory descriptors supported the good overall acceptability of traditional cheeses produced with ASCs and of the advanced traditional cheeses. A more accurate sensory analysis, subject to the appropriate statistical evaluation, should be carried out to refine production of both cheeses.

### 3.5. Persistence of Pro-Technological and Probiotic Strains

**Figure 6:**
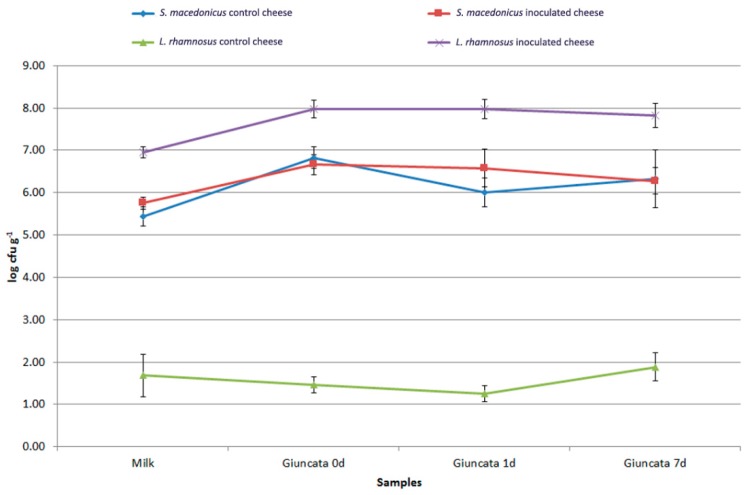
Population dynamics of *S.* *macedonicus* and *Lactobacillus rhamnosus* at different steps of the production and cold storage of uninoculated and inoculated advanced Giuncata Leccese samples.

Monitoring of both the pro-technological ASC2 starter and the probiotic *Lb. rhamnosus* LR04 strain from samples collected during different steps of the production process and cold storage of Giuncata cheese was carried out as reported in Section 2.2 and Section 2.3. A total of 54 colonies of lactic acid bacteria with coccus morphology and 47 colonies with rod morphology were isolated from the plates used for counting. The isolates were purified by the streaking technique and identified using molecular approaches [13,15,30]. Species-specific PCR of the isolates from the MMV (Di Lena et al., 2015) and SM [29] plates confirmed the presence of both the probiotic strain and the technological starter up to Day 7 of cold storage. In particular, the added probiotic strain accounted for approximately 10^8^ CFU/g in samples from the inoculated cheese, remaining quite constant up to the end of the shelf-life of the Giuncata (Figure 6). On the other hand, *Lb. rhamnosus* viable cells were absent in samples from the uninoculated Giuncata. *S. macedonicus* accounted for approximately 7 log_10_ CFU/g in both inoculated and uninoculated fresh cheeses, remaining stable in the inoculated Giuncata after one day (T1) of cold storage, and decreasing to 6 log CFU/g in the uninoculated Giuncata T1. *S. macedonicus* accounted for a little more than 6 log CFU/g in both cheeses after 7 days of cold storage (Figure 6). The PCR-based approach of Di Lena et al. [7] allowed us to confirm that the *Lactobacillus* counts registered in the inoculated cheese samples were attributable to the probiotic strain.

**Figure 7:**
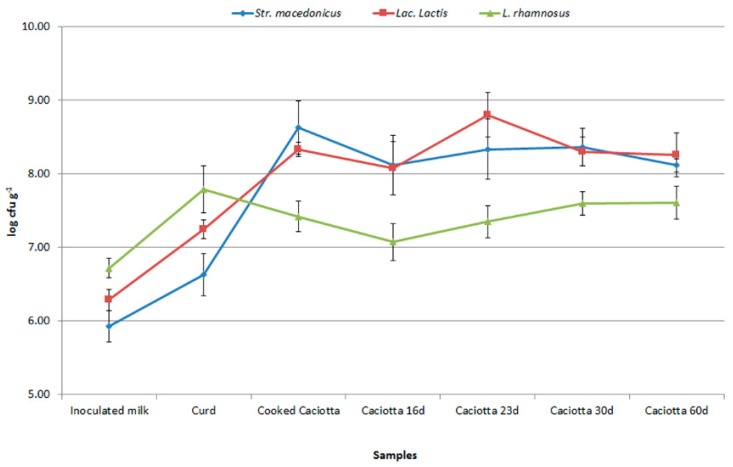
Population dynamics of *S. macedonicus*, *Lactococcus* spp. ASC4, and *Lb.* *rhamnosus* at different steps of production of the inoculated Caciotta.

The presence of the technological starter ASC4 (composed of *S. macedonicus* 62GT0 and *Lc. lactis* LC51) was confirmed by species-specific PCR over 60 days of ripening of Caciotta (both uninoculated and inoculated with the probiotic strain), reaching values higher than 10^8^ CFU/g (Figure 7). High counts of the probiotic *Lb. rhamnosus* LR04 were also registered until Day 60 in the cold-stored ripened samples. Based on these results, the protocol of cheese-making adopted in this work was suitable for manufacturing a ripened cheese containing high concentrations of the probiotic and indigenous strains.

### 3.6. Monitoring of the Probiotic Strain in Human Trials

In order to assess the ability of Caciotta to transport *Lb. rhamnosus* LR04 cells into the human gastrointestinal tract, 100 g of cheese, containing approximately 3 × 10^11^ CFU/g of the probiotic strain, was administered daily (intervention period, 10 days) to three healthy volunteers (two males and one female) aged 33 to 39 years, following the protocol described in Section 2.7 Microbiological analyses of fecal samples were performed by plating sample dilutions on MMV agar [7].

**Figure 8:**
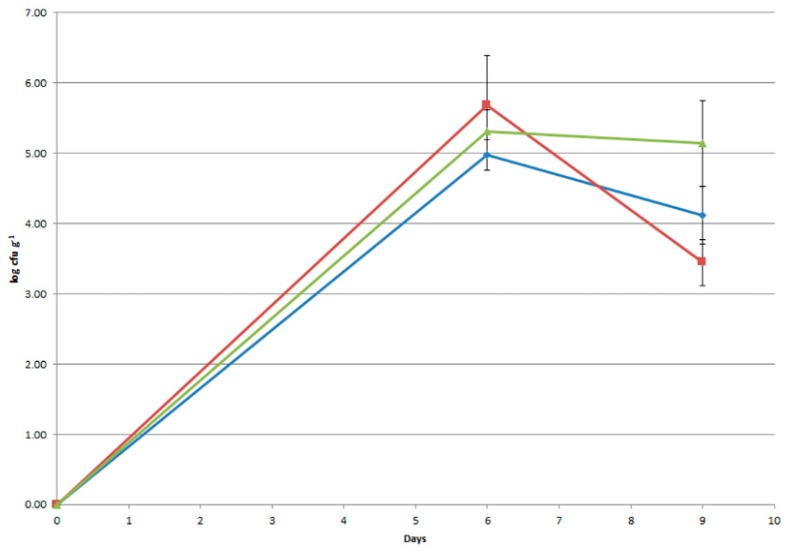
*Lb. rhamnosus* LR04 counts from the feces of three healthy volunteers (different colors) fed with Caciotta cheese containing the probiotic identified by species-specific and RAPD-PCR.

More than 70 colonies were isolated, purified, and subjected to DNA extraction, species-specific PCR [30], and REP- and RAPD-PCR [7], allowing description of the survival of *Lb rhamnosus* LR04 in human feces (Figure 8). At the beginning of administration, the feces of volunteer did not present viable microbial cells belonging to *Lb. rhamnosus.* After one week of supply, the count of *Lb. rhamnosus* strain in feces amounted from 9.57 × 10^4^ to 4.83 × 10^5^ CFU/g; the strain was present in the feces with a count of at least 2.79 × 10^3^ CFU/g even 3 days after the last administration (Figure 8).

## 4. Conclusions

The culture-dependent molecular approach used to study the microbiota of both Giuncata and Caciotta Leccese cheeses allowed isolation and identification of the lactic acid bacteria involved in their production among the microbial populations present in the different stages of cheese production and ripening. The selection of strains endowed with promising technological features allowed formulation of autochthonous starter cultures (ASC) used for manufacturing both cheeses at a pilot scale. ASC survived both cheese production processes and cold storage. A commercial probiotic strain was also employed to manufacture the advanced traditional cheeses; even though it was isolated from the human gastrointestinal tract, it was found to be able to adapt, grow, and persist up to the end of cold storage in both cheese matrices. The probiotic strain also survived in the feces of healthy volunteers fed with ripened cold-stored Caciotta even several days after the cheese intake, showing a good aptitude of this cheese as an effective carrier for the delivery of this probiotic adjunct to the human body. The combined inoculums of both the autochthonous starters and the probiotic strain for manufacturing advanced traditional Giuncata and Caciotta Leccese cheeses positively influenced the sensory characteristics of the final cheeses, and they were well appreciated by expert tasters. Overall, this study proposes the use of autochthonous starters in combination with a commercial probiotic strain to satisfy the industrial requirements of cheese manufacturers producing traditional dairy foods fortified with probiotic adjuncts.

To the best of our knowledge, this is the first study reporting the use of a *S. macedonicus* strain as an adjunct for the production of cheeses with added probiotics.

## Figures and Tables

**Table 1:** Occurrence of different species of lactic acid bacteria during cheese-making of Giuncata leccese cheese. Number of isolates from each sample with isolation dilution is reported in parentheses.

Bacterial Species	RM	PM	Samples ^1^					Total
			C	G	G5d	G12d	G20d	
*Lactococcus lactis*	6 (−3)		7 (−4)	6 (−3)	1(−3)	12 (−5)	4 (−6)	35
subsp. *lactis*								
*Lactobacillus casei*	1 (−3)							1
*Streptococcus parauberis*	1 (−3)			2 (−3)	10 (−3)	3( −5)	2 (−5)	18
*Streptococcus thermophilus*	1 (−3)	12 (−2)	3 (−5)	11 (−4)	10 (−3)	2 (−3)		39
*Streptococcus macedonicus*	2 (−3)	1 (−3)	3 (−6)	12 (−4)	11 (−3)	2 (−3)		31
*Leuconostoc mesenteroides*	1 (−3)						3 (−6)	4
subsp. *mesenteroides*								
*Enterococcus faecium*	1 (−3)			1 (−3)				2
*Streptococcus bovis*	1 (−3)			1 (−3)				2

^1^ Samples were collected during cheese-making and refrigeration of Giuncata leccese cheese. RM, raw whole cows’ milk; PM, pasteurized milk; C, curd; G, Giuncata cheese; G5d, G12d, and G20d, Giuncata cheese at 5, 12, and 20 days, respectively, of storage at 7 °C.

**Table 2:** Autochthonous lactic acid bacteria selected for further technological characterization.

Species	Strain	Source
*Lactococcus lactis*	L0 1-1	Caciotta 24 h after salting
	L0 1-6	Caciotta 24 h after salting
	L0 3-2	Caciotta 24 h after salting
	L0 3-5	Caciotta 24 h after salting
	C51	Giuncata curd
*Leuconostoc mesenteroides*	L0 3-4	Caciotta 24 h after salting
	L0 3-7	Caciotta 24 h after salting
	Lt1 3-1	20 days ripened Caciotta
	Lt1 3-2	20 days ripened Caciotta
	Lt1 3-15	20 days ripened Caciotta
*Streptococcus macedonicus*	Lt1 2-1	20 days ripened Caciotta
	Lt1 4-3	20 days ripened Caciotta
	Lt1 4-11	20 days ripened Caciotta
	B2-2	Thermized milk
	62Gt0	Ready produced Giuncata
*Streptococcus thermophilus*	Lt1 2-5	20 days ripened Caciotta
	B2-3	Thermized milk
*Lactobacillus delbrueckii*	Lt1 4-1	20 days ripened Caciotta
	Lt1 4-2	20 days ripened Caciotta
*Lactobacillus paracasei*	Lt1 3-11	20 days ripened Caciotta

**Table 3:** Counts (CFU/g) of *Lc. lactis* LC51 and *S. macedonicus* 62GT0 from mini-productions of Giuncata Leccese cheeses at a laboratory scale.

Trial	*S. macedonicus*	*Lc. lactis*
Control (C)		
Fresh Giuncata Leccese (T0) *	<100	<100
Fresh Giuncata Leccese (T1) **	<100	<100
Fresh Giuncata Leccese (T4) ***	<100	<100
*Str. macedonicus* 62GT0 (ASC2)		
Fresh Giuncata Leccese (T0)	6.95 × 10^6^	
Fresh Giuncata Leccese (T1)	1.37 × 10^7^	
Fresh Giuncata Leccese (T4)	4.95 × 10^7^	
*Lc. lactis* LC51 (ASC1)		
Fresh Giuncata Leccese (T0)		9.63 × 10^8^
Fresh Giuncata Leccese (T1)		2.30 × 10^9^
Fresh Giuncata Leccese (T4)		6.45 × 10^9^
*Lc. lactis* LC51 + *Str. macedonicus* 62GT0 (ASC3)		
Fresh Giuncata Leccese (T0)	1.30 × 10^7^	9.49 × 10^8^
Fresh Giuncata Leccese (T1)	2.32 × 10^7^	2.23 × 10^9^
Fresh Giuncata Leccese (T4)	8.14 × 10^7^	6.30 × 10^9^

* (T0) = Giuncata at time 0; ** (T1) = Giuncata after 1 day of cold storage; *** (T4) = Giuncata after 4 days of cold storage.

**Table 4:** Comparison between taste (flat, sweet, acidic, and bitter) and texture (creamy, soft, gummy, and hard) attributes of 48 h refrigerated Giuncata cheese samples with (ASC) and without (control cheese, CC) autochthonous starter culture.

Assessors	Sensory Descriptors															
	Flat		Sweet		Acidic		Bitter		Creamy		Soft		Gummy		Hard	
	CC	ASC	CC	ASC	CC	ASC	CC	ASC	CC	ASC	CC	ASC	CC	ASC	CC	ASC
1	±	-	±	±	-	±	±	-	+	+	+	+	-	-	-	-
2	-	-	±	-	-	-	-	-	+	+	+	+	-	-	-	-
3	±	-	±	±	-	±	±	-	+	+	+	+	-	-	-	-
4	-	-	±	±	-	-	-	-	+	+	+	+	-	-	-	-
5	±	-	+	-	-	±	±	-	+	+	+	+	-	-	-	-
6	±	±	+	-	±	+	-	-	+	+	+	+	-	-	-	-

−, not recognized; ±, weak; +, moderate; ++, strong.

**Table 5:** Comparison between taste (flat, sweet, acidic, and bitter) and texture (creamy, soft, gummy, and hard) attributes of two-month-ripened Caciotta cheese samples with (ASC) and without (control cheese, CC) autochthonous starter culture.

Assessors	Sensory Descriptors															
	Flat		Sweet		Acidic		Bitter		Creamy		Soft		Gummy		Hard	
	CC	ASC	CC	ASC	CC	ASC	CC	ASC	CC	ASC	CC	ASC	CC	ASC	CC	ASC
1	-	±	-	-	-	-	±	-	±	+	+	+	-	-	-	-
2	-	±	-	-	±	-	-	-	-	+	-	+	±	-	-	-
3	-	+	-	-	-	-	±	-	-	+	-	+	±	-	-	-
4	-	±	-	-	-	-	-	-	±	++	+	+	-	-	-	-
5	-	±	-	-	-	-	±	-	-	+	+	+	-	-	-	-
6	-	+	-	-	±	-	-	-	-	+	-	+	-	-	-	-

−, not recognized; ±, weak; +, moderate; ++, strong.
